# A Resected Case of Simultaneous Gastric and Multiple Pancreatic Metastases of Renal Cell Carcinoma 11 Years After Nephrectomy

**DOI:** 10.7759/cureus.69108

**Published:** 2024-09-10

**Authors:** Hideki Mori, Hiroki Sunagawa, Hirofumi Matsumoto

**Affiliations:** 1 Department of Gastroenterology, Nakagami Hospital, Okinawa, JPN; 2 Department of Surgery, Nakagami Hospital, Okinawa, JPN; 3 Department of Pathology, Nakagami Hospital, Okinawa, JPN

**Keywords:** gastric metastasis, late metastatic recurrence, pancreatic metastasis, renal cell carcinoma, total pancreatectomy

## Abstract

A 50-year-old man with a history of nephrectomy for renal cell carcinoma (RCC) 11 years prior was diagnosed with gastric and multiple pancreatic metastases of RCC. He underwent a pyloric gastrectomy and total pancreatic resection. RCC metastases to the pancreas are rare, and gastric metastases are even rarer. This case represents a rare instance of simultaneous RCC metastases to both the stomach and pancreas. Although there is no difference in prognosis between solitary and multiple pancreatic metastases, surgical resection is recommended even for multiple lesions. However, preoperative imaging often fails to identify all pancreatic metastatic lesions, making total pancreatectomy a consideration for ensuring complete resection, especially when preoperative detection is challenging.

## Introduction

Renal cell carcinoma (RCC) is the most common type of kidney cancer, originating in the lining of the renal tubules. RCC can metastasize, spreading to other organs either simultaneously (synchronous metastasis) or long after the primary tumor has been treated (metachronous metastasis) [1]. Complete resection of these metastatic lesions is recommended due to its associated survival benefit compared to no metastasectomy [2]. Pancreatic metastatic RCC (mRCC) is rare, with a prevalence of 1.4-2.8% [3], while gastric mRCC is even rarer, occurring in 0.2-0.7% of cases [4]. To date, only three published case reports have documented both gastric and pancreatic mRCC. The complete resection of pancreatic mRCC is particularly challenging because identifying all metastatic lesions preoperatively is difficult [5,6]. In fact, over 50% of cases pathologically confirmed as having multiple pancreatic mRCC lesions were evaluated as solitary lesions before surgery [7,8]. We report a case of simultaneous mRCC in the stomach and pancreas occurring 11 years after a left nephrectomy for RCC.

## Case presentation

**Figure 1:**
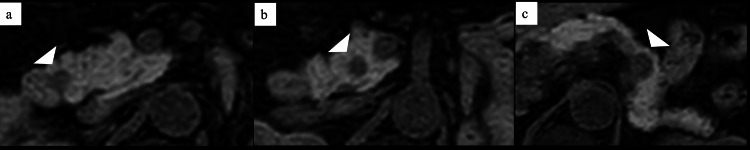
T1-weighted MRI with fat suppression T1-weighted MRI with fat suppression reveals two distinct low-signal nodules in the pancreatic head (a, b; arrowhead) and one in the pancreatic tail (c; arrowhead). These nodules were subsequently confirmed to be metastatic lesions from RCC. RCC, renal cell carcinoma

**Figure 2:**
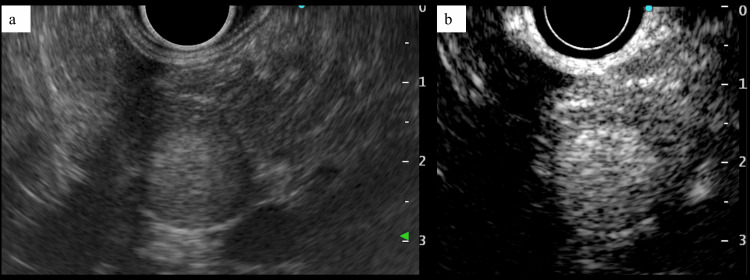
EUS and contrast-enhanced EUS (a) EUS shows a 12-mm mass in the pancreatic tail with higher echogenicity compared to the surrounding parenchyma. (b) Contrast-enhanced EUS reveals a homogeneous, hypervascular lesion in the pancreatic tail, consistent with mRCC, which typically presents as a hypervascular mass. EUS, endoscopic ultrasonography; mRCC, metastatic RCC

A 50-year-old man with a history of left nephrectomy for RCC 11 years prior was referred to our department after a pancreatic tumor was detected on regular follow-up contrast-enhanced CT. He had previously undergone left retroperitoneoscopic nephrectomy and adrenalectomy for a left renal tumor (clear cell RCC, pT1aN0M0, stage I [International Union Against Cancer Manual, Eighth Edition]). Physical examination was normal. Laboratory tests, including pancreatic enzymes and tumor markers, revealed only mild renal impairment. CT scans identified an 11 mm enhanced nodule in the pancreatic head. T1-weighted MRI with fat suppression showed two distinct low-signal nodules in the pancreatic head and one in the pancreatic tail (Figure 1). These nodules demonstrated enhancement on arterial-phase MRI but were difficult to detect on diffusion-weighted images. Endoscopic ultrasonography (EUS) revealed two 6 mm well-defined, circular masses in the pancreatic head and one 12 mm mass in the pancreatic tail, which had higher echogenicity compared to the surrounding parenchyma (Figure 2). The lesion in the pancreatic tail appeared homogeneous and hypervascular in both the early and late phases of contrast-enhanced EUS (Figure 2).

**Figure 3:**
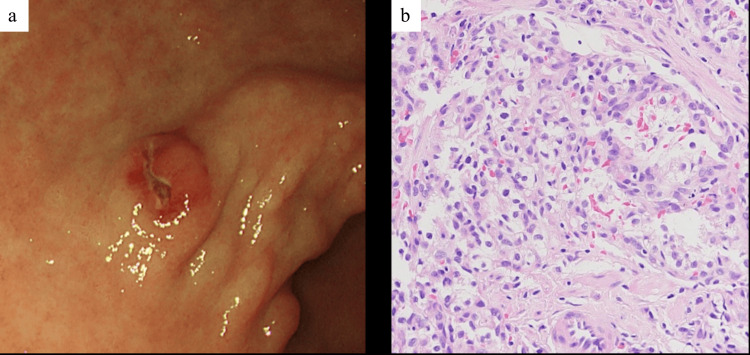
Endoscopic image of the gastric body and the corresponding pathological findings from the biopsy specimen (a) EGD reveals an SMT with central apical erythema and erosion on the anterior wall of the lower gastric body. (b) H&E staining of the biopsy shows atypical cells with small round nuclei and clear cytoplasm, consistent with mRCC. EGD, esophagogastroduodenoscopy; mRCC, metastatic RCC; SMT, submucosal tumor

Esophagogastroduodenoscopy screening revealed a submucosal tumor (SMT) with central apical erythema and erosion on the anterior wall of the lower gastric body (Figure 3), which was undetectable on CT. A biopsy of the SMT showed atypical cells with small round nuclei and clear cytoplasm on H&E staining (Figure 3). These cells were positive for CD10 and CKAE1/AE3, indicating RCC gastric metastasis.

**Figure 4:**
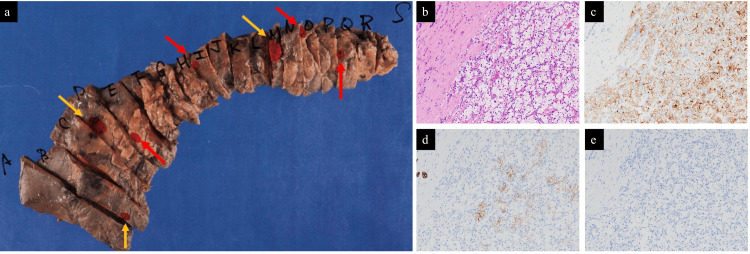
Macroscopic and pathological findings of the resected specimens (a) Pathological examination of the pancreas reveals seven lesions. Three lesions were identified preoperatively (yellow arrows), while four lesions were not detected preoperatively (red arrows). (b) H&E staining shows atypical cell proliferation with small round nuclei and clear cytoplasm. Immunohistochemistry shows tumors positive for CD10 (c), sporadically weakly positive for CK7 (d), and negative for CK20 (e).

Fluorodeoxyglucose-PET (FDG-PET) showed no accumulation in the gastric body, pancreas, or other organs, including the lung, liver, and bone. Given the preoperative diagnosis of gastric and multiple pancreatic mRCC, pyloric gastrectomy and total pancreatic resection were performed. Pathological examination of the pancreas revealed seven lesions: three in the pancreatic head, one in the pancreatic body, and three in the pancreatic tail (Figure 4). H&E staining showed atypical cell proliferation with small, round nuclei and clear cytoplasm (Figure 4). Immunohistochemical analysis confirmed that the tumors were positive for CD10, sporadically weakly positive for CK7, and negative for CK20, consistent with multiple pancreatic mRCC (Figure 4). Four lesions (one in the pancreatic head, one in the pancreatic body, and two in the pancreatic tail) were not identified even after a postoperative imaging review. Pathological examination of the stomach identified a single lesion with atypical cells having round nuclei and clear cytoplasm, consistent with gastric mRCC. The tumor cells had partially invaded the submucosa, but no lymph node metastasis was observed. The pathological findings confirmed one gastric and multiple pancreatic mRCC. The postoperative course was uneventful, and the patient has remained tumor-free for one year following surgery.

## Discussion

The recurrence rate after surgical resection of primary RCC is approximately 30% [9], with hematogenous metastasis commonly occurring in organs such as the lung, liver, and bone. Only three case reports have documented both gastric and pancreatic mRCC to date (Table 1) [10-12].

**Table 1:** Cases of pancreatic and gastric metastases of RCC ESD, endoscopic submucosal dissection; DP, distal pancreatectomy; GM, gastric metastasis; PD, pancreaticoduodenectomy; PM, pancreatic metastasis; RCC, renal cell carcinoma; TP, total pancreatectomy

Reference	RCC stage	Diagnosis of PM	Treatment	Diagnosis of GM	Treatment
[10]	Unknown	10 years later	DP	11 years later	Total gastrectomy for bleeding control
[11]	Unknown	21 years later	TP	19 years later	Partial gastrectomy
[12]	pT1N0M0	10 years later	PD	12 years later	ESD
Our case	pT1N0M0	11 years later	TP	11 years later	Partial gastrectomy

In a systematic review, complete resection of mRCC demonstrated a survival benefit compared to not performing metastasectomy [2]. For cases with multiple organ metastases, the five-year cancer-specific survival rate was higher in patients who underwent complete resection compared to those who did not receive metastasectomy [13]. Among patients with gastric mRCC, 31 out of 54 underwent surgical or endoscopic resection, with many surviving without recurrence following the procedure [14]. For pancreatic mRCC, the five-year survival rate was 72.6% for resected cases compared to 14% for non-resected cases [15]. Although pancreatic total resection was performed in 19.9% of cases with four or more pancreatic metastases (21.2% of resected cases), overall survival did not significantly differ between solitary and multiple lesions or between partial and total pancreatectomy cases [15].

Recent advancements in molecularly targeted therapies for mRCC have shown that surgical resection of mRCC to the pancreas might not always improve survival compared to tyrosine kinase inhibitor therapy [16]. However, it has been noted that surgical resection can achieve disease-free status in some patients. Recent studies indicate that even with multiple pancreatic metastases, where 69% of cases have multiple pancreatic metastases and 54% have additional extra-pancreatic metastases, surgical treatment alone has shown promising results in terms of both survival and quality of life [17]. Consequently, surgical resection is recommended for pancreatic mRCC, even in cases with multiple metastases [17,18].

Despite numerous case reports of multiple pancreatic mRCCs, detecting all pancreatic metastatic lesions preoperatively remains challenging. Small pancreatic mRCCs are often iso-dense even after CT enhancement, making them difficult to detect [5]. Additionally, pancreatic mRCCs may show reduced FDG uptake on FDG-PET, as observed in our patient, where no FDG accumulation was detected [6]. Preoperatively, 50-62% of cases pathologically confirmed as having multiple pancreatic mRCC lesions are often evaluated as solitary lesions, highlighting the limitations of current imaging techniques in detecting multiple metastases [7,8].

In our case, four out of seven pancreatic lesions were not identified preoperatively, underscoring the difficulty in detecting small or iso-dense metastases. These findings illustrate the limitations of current imaging modalities and their potential impact on surgical planning. Among patients undergoing pancreatectomy for pancreatic mRCC, 33-42% experience residual pancreatic recurrence, likely due to undetected micrometastases [8,19]. Therefore, the risk of residual micrometastases should be carefully considered when deciding on local resection, with total pancreatectomy being the preferred approach for patients who can tolerate the surgery.

## Conclusions

Pancreatic metastasis from RCC is rare, and gastric metastasis is even rarer. This case involves simultaneous mRCC to both the stomach and pancreas, occurring 11 years after nephrectomy. It was managed with pyloric gastrectomy and total pancreatectomy. Surgery is recommended for RCC metastases if complete resection is feasible. Identifying multiple pancreatic metastases preoperatively is challenging, and the risk of residual micrometastases should be considered when opting for local resection. Total pancreatectomy is recommended as the preferred approach for young, compliant patients.
